# Impact of educational intervention on understanding health recommendations after liver transplantation

**DOI:** 10.1590/0034-7167-2023-0132

**Published:** 2024-07-29

**Authors:** Josely Santana do Amorim, Angela Aparecida de Lima, Agnaldo Soares Lima

**Affiliations:** I Universidade Federal de Minas Gerais, Hospital das Clínicas. Belo Horizonte, Minas Gerais, Brazil; II Universidade Federal de Minas Gerais, Programa de Pós-Graduação em Cirurgia e Oftalmologia. Belo Horizonte, Minas Gerais, Brazil.

**Keywords:** Health Education, Liver Transplantation, Patient Discharge, Comprehension, Self Care, Educación en Salud, Trasplante de Hígado, Alta del Paciente, Comprensión, Autocuidado

## Abstract

**Objectives:**

to evaluate the impact of educational intervention on understanding health recommendations after liver transplantation.

**Methods:**

randomized and prospective clinical trial, with 68 liver transplant recipients in two institutions. The level of understanding was assessed using a statement agreement scale and the understanding score was classified. Chi-square test was used to compare groups.

**Results:**

the level of understanding was reasonable in 77.9% of patients, 73.5% in the Control Group and 82.3% in the Intervention Group (p=0.399). For topics covered after educational action, there were more than 80% correct answers regarding nutrition, frequent hydration, usage and function of immunosuppressants. However, there were less than 10% correct answers regarding hand hygiene, contact with animals and crowds of people. The use of the patient’s audio, visual and tactile resources led to improved understanding of skin care (p=0.014).

**Conclusions:**

the level of understanding acquired regarding health recommendations was only reasonable.

## RESUMEN

**Objetivos:**

evaluar el impacto de una intervención educativa sobre la comprensión de las recomendaciones sanitarias tras un trasplante de hígado.

**Métodos:**

se trata de un ensayo clínico prospectivo aleatorizado con 68 pacientes trasplantados de hígado de dos instituciones. Se evaluó el nivel de conocimiento mediante una escala de acuerdo con las afirmaciones y se clasificó la puntuación de la comprensión. Se utilizó la prueba de chi-cuadrado para comparar los grupos.

**Resultados:**

el nivel de comprensión fue razonable en el 77,9% de los pacientes, con el 73,5% del Grupo de Control y el 82,3% del Grupo de Intervención (p=0,399). En cuanto a los temas tratados tras la actividad educativa, más del 80% tenía conocimiento sobre alimentación, hidratación frecuente, uso y función de los inmunosupresores. Sin embargo, hubo menos de un 10% de respuestas correctas sobre la higiene de las manos, el contacto con animales y las aglomeraciones de personas. La utilización de los recursos auditivos, visuales y táctiles del paciente mejoró la comprensión del cuidado de la piel (p=0,014).

**Conclusiones:**

el nivel de comprensión adquirido sobre las recomendaciones sanitarias fue solo razonable.

## INTRODUCTION

Health education is considered one of the paths that favors health promotion, through educational interventions that help in the development of individual responsibility and disease prevention. These actions are outlined by health professionals in order to enable the patient not only to understand their health status, but also to enable them to make decisions related to their health care^(1)^.

The nurse can be considered a catalyst for change to promote health, when carrying out educational interventions with patients. Catalyzing changes means creating environments and configurations of educational interventions that strengthen the participation and ownership of individuals, acting as a leader of their own treatment^(2)^. In nursing, these practices direct individual training, aim to facilitate and develop personal skills, which consequently result in self-care empowerment, in addition to strengthening the bond between patient and professional^(2-3)^.

Educational intervention, whether individual or in groups, provided in an interactive way promotes learning and represents great potential to promote adherence to the desired behavior in treatment. To achieve this, the chosen educational strategy must be based on empowering the person, in order to make them responsible for managing their health situation, supporting adherence as a focus for the practice of self-care^(3)^. Learning is defined as the process that allows individuals to modify their behavior^(1)^.

Liver transplantation is a complex surgical procedure that involves permanent care due to the risk of graft rejection. To achieve this, it is necessary to adhere to lifestyle changes, such as strict medication use and monitoring, hygienic habits, oral hydration and preventive measures for infection^(1)^.

After liver transplantation, the individual needs knowledge to change some habits and improve their quality of life. This behavioral change includes adherence to the proposed treatment after the transplant, which involves the new medication regimen, attending appointments with the health team, performing routine physical activity and paying attention to eating habits. It is crucial that patients take responsibility for their treatment. Lack of knowledge for the conduction of the recommendations to be practiced has a negative influence on the process of adherence to treatment, becoming a risk for the evolution of adequate graft functioning.

## OBJECTIVES

To evaluate the impact of an educational intervention on understanding health recommendations after liver transplantation. The following hypothesis was tested: Does an educational intervention have a positive impact on patient’s understanding health recommendations after liver transplantation?

## METHODS

### Ethical aspects

The present study was approved by the Research Ethics Committees from the Federal University of Minas Gerais and Santa Casa in Belo Horizonte, registered in the Brazilian Clinical Trials Registry (ReBEC), under the code RBR-454t52.

### Study Design

This is a randomized and prospective clinical trial. This study followed the Consolidated Standards of Reporting Trials (CONSORT)^(4)^ guidelines for the presentation of clinical trials and was registered in the Brazilian Registry of Clinical Trials (REBEC) under the registration code RBR-454t52.

### Period, setting, and population

The study was carried out, from January 2016 to July 2019, in two general and public hospitals in Belo Horizonte that have a liver transplant program registered in the National Transplant System. Patients aged 18 years old or over, hospitalized for their first liver transplant, with clinical conditions to receive health recommendations for home self-care after hospital discharge, were included.

### Sample selection and criteria

**Figure 1:**
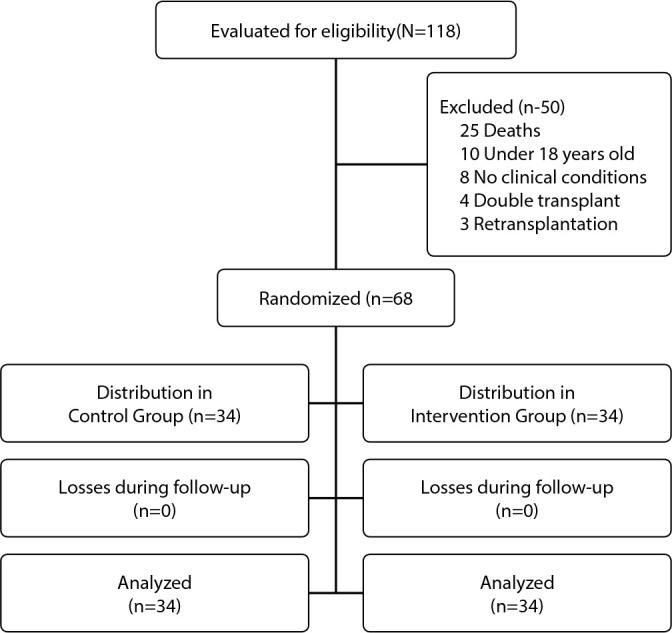
Flowchart of study sample formation

Randomization occurred on the day of transplantation through a draw. Recruitment was carried out by the researcher after the transplant, one day after transferring the patient from the intensive care unit to the hospitalization ward. In the initial approach, patients who agreed to participate in the educational action signed the informed consent form and were included in the study. Informed Consent was obtained from all individuals involved in the study in writing. The sample size, determined using a significance level of 5% and an estimated proportion of 0.5, indicated the need for at least 31 patients/group. In total, 118 patients underwent liver transplantation in these healthcare institutions during the study period. Of these transplant recipients, 68 met the inclusion criteria and were distributed to one of the two groups of the study: those who received health recommendations plus specific recommendations on immunosuppressive drugs, using the patient’s visual, auditory and tactile resources in the educational action, were allocated to the intervention group (IG; n=34), and the control group (CG; n=34) was composed of those who received health recommendations using the patient’s auditory and visual resources in the conventional educational action, consolidated in the institution. Figure 1 shows the CONSORT diagram, with the flow of participants during the study phases.

### Instruments

In the conventional educational action of the CG, an explanatory and validated form was used in health institutions, with consolidated use for hospital discharge of liver transplant patients. For the IG, material prepared by the author was used (educational folder and spiral binding of 15 illustrative figures in JPEG format printed on A3 sheets), not revalidated as it was the reorganization of information from the institutional printout added with attractive colors, figures and details.

### Intervention

The educational action took place at the time of hospital discharge, when each of the transplant patients received the same specific health recommendations for self-care. These were provided in different ways for the IG and the CC. The health recommendations included nutrition, taking medications at the correct times and doses, and not taking the dose before carrying out laboratory serum tests to control the dosage of immunosuppressants. Behaviors to be avoided were also recommended, such as crowding of people, contact with animals, contact with newborns who were immunized with the Sabin vaccine and with sick people. Furthermore, wearing a mask in the first 2 months post-transplant, practicing light physical exercise such as walking, drinking water frequently, applying moisturizing cream daily and attending outpatient appointments on the stipulated dates were also emphasized.

**Figure 2:**
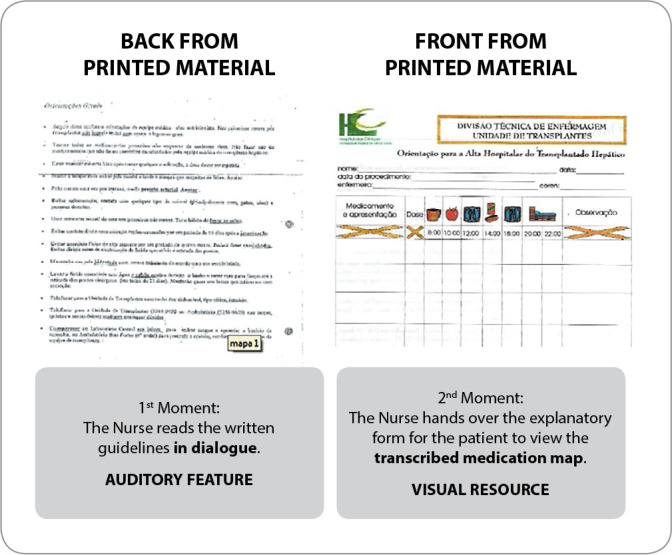
Graphic piece used in the Control Group’s educational action

The educational action was carried out as follows in the groups: The CG received recommendations for hospital discharge from the nurses who were members of the Nursing Team of the hospitalization ward of the health institutions under study. The material used was an explanatory printout specific to health institutions, containing a page printed on both sides. On the front there were instructions for the medication map, illustrated with medication taking times, and on the back there were written health recommendations, as shown in Figure 2.

**Figure 3:**
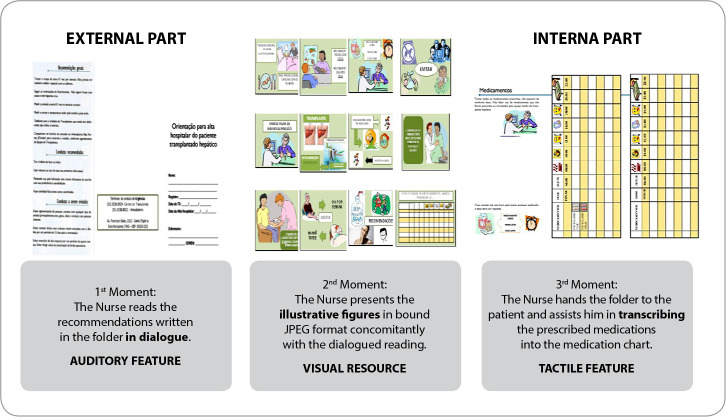
Graphic piece used in the Intervention Group’s educational action

The operationalization of the CG’s educational action began with the nurse transcribing the medications prescribed by the medical team into the medication map. Then, the professional went to the patient’s room and carried out the educational action in two moments, as shown in Figure 2. In the first moment, the patient’s auditory resource was used, with the nurse carrying out a dialogued reading of the recommendations written on the back of the printed form. In the second moment, the patient’s visual aid was used with the delivery of a printed form with a transcribed medication map. The nurse, together with the patient, checked the medications patients’ had in their hands with those prescribed, identified them on the map of transcribed medications and showed the doses to be administered by the patient at home. The IG received the recommendations from the research nurse, with material used that included a printed folder plus a medication map and bound material with illustrative figures corresponding to all health recommendations and specific immunosuppressive medications, according to Figure 3.

The operationalization of the IG’s educational action took place in three moments using the patient’s auditory, visual and tactile resources, according to Figure 3. The first and second moments took place concomitantly, using the patient’s auditory resources with dialogued reading of the educational folder and visual resource for the patient with presentation of bound illustrative figures referring to the recommendations in the folder. In the third moment, the patient’s tactile resources were used to manipulate the medications in order to recognize them (shape and texture) followed by the creation of the map contained in the folder. With the help of the nurse, the patient transcribed the medications prescribed by the doctor, as well as marking the times and doses to be administered. The professional also helped the patient identify the medications in hand and their location on the map transcribed by him.

### Data collection

Data collection took place 15 days after hospital discharge, in the outpatient clinic, where the patient was interviewed. Two questionnaires prepared by the author were used, the first with information on the sociodemographic and clinical variables described in the literature as relevant (age, gender, city of residence, educational background, years of study, indication for transplant, date of registration on the waiting list, date of transplant and date of hospital discharge), completed by the researcher through an interview and consultation of medical records. The second questionnaire aimed to estimate understanding of the recommendations and was composed of 13 multiple-choice questions, referring to the recommendations received during hospital discharge. This questionnaire was given to the participant to be completed (self-administered) by an external evaluator, who had no knowledge about the educational actions and objectives of the study. For each statement, the interviewee indicated only one answer, such as: totally true, partially true, can’t say, partially false and totally false. The script assessed the patient’s level of understanding from health recommendations received during hospital discharge and experienced for 15 days at home. The total score indicates the number of items that were answered correctly (1 point for each correct answer), making it possible to obtain points from 0 to 13. After summing up the points obtained, an analysis of agreement was carried out between the interviewee’s response and the guidance provided during hospital discharge. The level of understanding was then classified as follows: Excellent, for interviewees who obtained 10 to 13 points, reasonable, for those who obtained 6 to 9 points, poor, for those who obtained 5 to 3 points, and very poor, for those below 2 points. In addition to classifying the interviewee’s level of understanding, there was also an investigation, at the end of the questionnaire, in relation to their opinion on the impact of the recommendations received during hospital discharge, in meeting their needs at home. This occurred through the answer to a perceptual multiple-choice question that classified recommendations received as insufficient, adequate or excellent. Each interviewee received a code to guarantee their anonymity in the study.

### Data processing and analysis

Statistical analysis was performed using the Software Package for Social Science (SPSS) version 20.0 for Mac (SPSS Inc., Chicago, Illinois, USA). Initially, a descriptive analysis of the data was carried out using frequency tables for nominal variables and those related to quantitative variables, which were summarized in the form of mean, median, standard deviation, interquartile and percentages. Then, this Kolmorov-Smirnov was applied to the distribution of quantitative variables. In the second moment, the Chi-square test was performed to compare CG and IG for nominal variables. The Chi-square test compared understanding and detected significant differences between nominal variables. The Student’s T test was performed for data with normal distribution and the Mann-Whitney test for data with non-normal distribution. The results were presented with mean plus or minus (±) standard deviation for numerical variables with normal distribution, and median and interquartile range for numerical variables with non-normal distribution. The data was distributed and related in tables.

## RESULTS

Among the 68 patients who participated in the study, 46 (67.6%) were men and 22 (32.4%) were women. Their median age at transplantation was 56.5 years (IIQ = 63.75). Thirty-seven patients (54.4%) came from the metropolitan region of Belo Horizonte and 31 (45.6%) came from inner cities from the State of Minas Gerais. The average number of years of study was 8.5 (±4.0), with 34 (50.0%) having completed primary school and 34 (50.0%) having secondary education or more. The average hospitalization time (from the day of transplantation to the day of hospital discharge) was 20 days (±12.6), with the median waiting time for an organ being 63.0 days (IIQ = 120 ,0). The indications for transplantation were grouped, resulting in 32 (47.1%) parenchymal liver disease, 19 (27.9%) neoplasia, 5 (7.4%) disease of biliary origin, 3 (4.4%) liver disease, autoimmune origin, 1 (1.5%) fulminant hepatitis and 8 (11.8%) other diseases were combined due to lack of similarity with diseases from previously established groups, namely: 02 Caroli’s disease, 03 Budd Chiari Syndrome, 01 Familial amyloidosis, 01 Alpha 1 Antitrypsin Deficiency and 01 Hereditary hemochromatosis.

Regarding the sample’s correct answer rates, the recommendations provided regarding: nutrition, correct use of immunosuppressant medication, functionality of immunosuppressants and recommendation of frequent oral hydration showed more than 80% of correct answers. Hand hygiene and behaviors to be avoided such as crowds and contact with animals expressed less than 10% of correct answers (Table 1).

**Table 1:** Assessment of the questionnaire on understanding the health guidelines provided at the time of hospital discharge from January 2016 to July 2019, Belo Horizonte, Minas Gerais, Brazil

Provided questions about the guidance	Sa† (n=68)		||CG (n=34)		¶IG (n=34)		*p* value^*^
	A‡ n, %	E§ n, %	A‡ n, %	E§ n, %	A‡ n, %	E§ n, %	
1) You must follow the nutritionist’s instructions, remembering that you cannot eat fruit with the skin or eat raw vegetables.	63 92.6	5 7.4	33 97.0	1 3.0	30 88.3	4 11.7	*0.163*
2) You must always wash your hands and rub them with alcohol.	4 5.9	64 94.1	1 3.0	33 97.0	3 8.9	31 91.1	*0.303*
3) You must work out twice a week in the first three months after the transplant.	38 55.9	30 44.1	17 50.0	17 50.0	21 61.7	13 38.3	*0.329*
4) You must take all the right medications with the correct dose and at the correct time as prescribed.	67 98,5	1 1.5	33 97.0	1 3.0	34 100	0 -	*0.314*
5) Before doing blood tests, you can take all medications.	37 54.4	31 45.6	17 50.0	17 50.0	20 58.8	14 41.2	*0.465*
6) You know that ISS^**^ medicines serve to prevent the destruction of the transplanted organ by the body’s defense.	63 92.6	5 7.4	31 91.1	3 8.9	32 94.1	2 5.9	*0.642*
7) ISS^**^ medications can cause increased glucose levels, headache, tremors, insomnia and diarrhea. If this happens, you must inform us during your consultations.	53 77.9	15 22.1	27 79.4	7 20.6	26 76.4	8 23.6	*0.770*
8) You should avoid closed places with lots of people.	4 5.9	64 94.1	1 3.0	33 97	3 8.9	31 91.1	*0.303*
9) Contact with newborn children after the Sabin vaccine and sick people is permitted.	47 69.1	21 30.9	21 61.7	13 38.3	26 76.4	8 23.6	*0.189*
10) It is advisable that you have help to take care of animals, especially cats, dogs and birds, as long as you do not touch them.	6 8.8	62 91.2	4 11.7	30 88.3	2 5.9	32 94.2	*0.393*
11) It is important that you wear a mask at all times.	27 39.7	41 60.3	13 38.2	21 61.8	14 41.2	20 58.8	*0.804*
12) It is recommended that you drink plenty of fluids, approximately 3 liters per day.	59 86.8	9 13.2	28 82.4	6 17.6	31 91.1	3 8.9	*0.283*
13) To keep your skin hydrated, you can use cream daily.	28 41.2	40 58.8	9 26.4	25 73.5	19 55.8	15 44.2	*0.014*

* Chi-square test; †Sa - Sample; ‡ A - Correct; § E - Error; ||CG - Control Group; IG - Intervention Group;

** ISS - Immunosuppressants.

In the comparative analysis of correct answers between the groups under study, participants from the CG and IG obtained more than 80% correct answers on questions relating to nutrition, correct usage of immunosuppressive medication, function of immunosuppressive medication and oral hydration. The IG participants obtained more correct answers, between 40% and 70%, in the recommendations regarding physical activity, not taking immunosuppressant medication before blood collection and behaviors to be avoided such as: contact with sick people and newborns vaccinated with Sabin, in addition to skin care. The skin care recommendation was answered incorrectly by 40 (58.8%) of the 68 study participants. In the comparison between the groups, only this recommendation showed a statistical difference (p = 0.014), with the most frequent correct answers being in the IG (Table 1).

Regarding the comparative analysis of incorrect answers between the groups, although the CG made more errors when questioning about hand hygiene recommendations and the avoidance of crowds of people, the IG was more frequently wrong about the avoidance of contact with animals, there was no significant difference in responses between the groups studied (Table 1).

Regarding the analysis of agreement between the interviewee’s response to the recommendations provided during hospital discharge of the 68 patients, 53 (77.9%) showed a reasonable level of understanding. 66 of them (97.0%) considered the information received to be excellent and adequate (Table 2).

**Table 2:** Comparative analysis between the Control Group and the Intervention Group of the participants’ level of understanding and opinion from January 2016 to July 2019, Belo Horizonte, Minas Gerais, Brazil

Variables	Categories	Sample n = 68 (n, %)	Control Grup n=34 (n, %)	Intervention Grup n=34 (n, %)	*p* value^*^
Participants' level of understanding of the instructions received	Excellent Reasonable Poor	03 (4.5) 53 (77.9) 12 (17.6)	01 (3.0) 25 (73.5) 08 (23.5)	02 (5.9) 28 (82.3) 04 (11.8)	0.399
Patient's opinion about the guidance received	Adequate Excellent Insufficient	19 (27.9) 47 (69.1) 02 (3.0)	07 (20.6) 26 (76.4) 01 (3.0)	12 (35.3) 21 (61.7) 01 (3.0)	0.397

* *Chi-square test.*

There was no significant difference in the level of understanding when comparing the study groups (p=0.399) (Table 2).

## DISCUSSION

The educational action took place with teaching resources and presentation of the material in different ways. This intervention took place during the hospital discharge of patients who underwent liver transplantation, providing an enlightening moment for the patient and their families. The findings of this study draw attention to the varied impact on participants’ understanding, evidenced by responses with extreme rates of errors and successes.

A positive impact can be seen from dietary recommendations, frequent oral hydration, functionality and use of immunosuppressants, which stood out due to the high rates of correct answers obtained. It is important to note that these recommendations were experienced by the patient during the hospital stay (which lasted an average of 20 days) and, consequently, were topics addressed daily by the healthcare team. Therefore, prior contact may have facilitated the understanding and maintenance of behaviors at home. However, the recommendation on hand hygiene did not have a relevant impact based on the responses obtained. Possibly, the frequent use of alcohol in the hospital environment may have caused some confusion in the patient about the correct way to wash their hands at home.

Changes in the educational action regarding this recommendation during health education may be necessary, to reinforce the habit of washing hands with soap and water, both for the patient and also for family and visitors. During the hospitalization period, the desire for improved health may arise, which causes the patient to create expectations that directly influence their recovery^(5)^. People are able to store much more information when they see and hear it. Repetition of information is cited as an important form of memorization, which leads us to rethink teaching-learning strategies currently used^(6)^.

Other recommendations, such as avoiding crowds of people and contact with animals (mainly cats, dogs and birds), had no impact, evidenced by lower accuracy rates among participants. It was observed that in these two topics it is also necessary to modify the way of teaching, reinforcing with examples of everyday situations that should be avoided, such as attending religious temples, stadiums, supermarkets, shopping malls, bank branches or sharing a bed or sofa with a domestic animal. It is also necessary to emphasize negative aspects of proximity to animals, such as the possibility of transmitting zoonoses through contact with the animal’s feces and saliva^(7)^.

In the present study, a relevant point was the positive impact on the group that received recommendations using the association of the patient’s auditory, visual and tactile resources, which reached the level of understanding of 82.3% of these participants. In this group, a better understanding of the recommendation for daily skin care with the application of moisturizing cream stands out (p=0.014).

In Spain, a study showed that educational measures after liver transplantation through the association of verbal, written and audiovisual resources as a teaching-learning strategy about healthy lifestyle habits were beneficial, corroborating the present study. According to the authors, the association of verbal, written and audiovisual resources in educational action is more effective than just the use of verbal resources^(8)^.

However, the present study detected a deficiency in understanding the skin care routine, since 58.8% of patients answered incorrectly about the daily use of moisturizing cream on their skin. Dermatological care is recommended because organ transplant recipients are at higher risk for infections and skin cancer. The immunosuppressive regimen alleviates about 70% of serious skin infections that appear during the first 3 months after transplantation. It is recommended that solid organ transplant recipients play an active role in preventing skin complications. Educational interventions can result in better information for this specific population and favor immediate treatment, with better clinical results^(9)^.

Regarding the prevention of skin cancer, a study showed that there is a tendency for men not to apply sunscreen to their skin routinely and regularly as self-care. Women use sunscreen more frequently on a daily basis than men^(10)^. This behavior reveals a problem to be combatted through elaborate campaigns for photo protection, with the aim of reaching the male public more efficiently and encouraging the habit of applying sunscreen^(11)^. Men seem less concerned about the harmful effects of ultraviolet radiation than women, who wear sunscreen more frequently, probably due to a greater sense of care for their health and aesthetics^(12)^.

Thus, if on the one hand the results pointed to a better understanding of the dermatological self-care of the IG participants, on the other hand, this skin care habit, which differs between genders in our society, draws attention. Knowledge of such behavior relevant for formulating concise, robust interventions targeted at this identified demand.

The evaluation of the educational action identified a reasonable level of understanding of the participants in the present study when using teaching material with explanatory and illustrated folders, explanatory printed material, medication map, in addition to the presence of the professional educator (nurse), who used auditory, visual and sensory senses of the patient to perform this action. The nurse plays a fundamental role in implementing these educational actions, which constitute relatively simple and low-cost, yet effective measures^(13)^. On topics that did not reach adequate levels of understanding, the study was useful in identifying the need for different approaches. The literature suggests the incorporation of the use of technologies for educational intervention, which can be an option used by transplant centers in a useful and effective way^(14-15)^. The use of applications for patient education is recent, however the use of messages, videos, images and animations are resources that present effective intervention results in terms of knowledge and care^(16)^.

As it was an educational intervention, it was expected that the educational level could compromise the patient’s understanding of the recommendations provided. The study showed that 77.9% of participants showed reasonable understanding, regardless of their level of education. Other studies corroborate this finding, also pointing out that individual perception and satisfaction are more influential in adherence to treatment than the level of education^(17-18)^.

This study showed that 69.1% of participants rated the recommendations given as excellent in meeting their needs. Even with the participants’ reasonable level of understanding, this finding reflects the interviewees’ state of satisfaction with the information that supported their home self-care.

### Study limitations

However, it is important to highlight that the present study has as a limitation: the lack of longitudinal results. However, this limitation did not compromise the quality of the evaluation and the results obtained, which aimed to verify the early impact on compliance with the recommendations.

### Contributions to the field

Our most relevant contribution was demonstrating that preparation for patient self-care can be carried out during the hospitalization period, with the participation of the assistant healthcare team, providing opportunities for the experience experienced by the patient and family.

We believe that the greater the number of approaches the nurse implements in the patient and family educational process, in its different forms, the better the continuity of self-care will be. However, to more adequately measure this effect, other specific prospective studies must be carried out.

## CONCLUSIONS

The educational health teaching action, carried out at the time of hospital discharge and focusing on the practice of self-care, achieved a reasonable level of understanding among transplant patients. The educational intervention developed had a positive impact on understanding health recommendations after liver transplantation.
