# Supplementary material for: Impact of educational intervention on understanding health recommendations after liver transplantation
Source: Rev Bras Enferm. 2024 Jul 29;77(3):e20230132. doi: 10.1590/0034-7167-2023-0132 (PMC11290744; doi:10.1590/0034-7167-2023-0132)
Supplement: Translation

# Translation: Impacto de intervenção educativa na compreensão sobre recomendações para saúde após transplante de fígado

**DOI:** 10.1590/0034-7167-2023-0132pt

**Authors:** Josely Santana do Amorim, Angela Aparecida de Lima, Agnaldo Soares Lima

**Affiliations:** I Universidade Federal de Minas Gerais, Hospital das Clínicas. Belo Horizonte, Minas Gerais, Brasil; II Universidade Federal de Minas Gerais, Programa de Pós-Graduação em Cirurgia e Oftalmologia. Belo Horizonte, Minas Gerais, Brasil.

**Keywords:** Educação em Saúde, Transplante de Fígado, Alta do Paciente, Compreensão, Autocuidado

## Abstract

**Objetivos:**

avaliar o impacto de intervenção educativa na compreensão sobre recomendações para saúde após transplante de fígado.

**Métodos:**

ensaio clínico randomizado prospectivo, com 68 transplantados de fígado em duas instituições. Avaliou-se o nível de compreensão por meio de escala de concordância de afirmativas e classificou-se o escore de compreensão. Utilizou-se teste qui-quadrado para comparação dos grupos.

**Resultados:**

o nível de compreensão foi razoável em 77,9% dos pacientes, sendo 73,5% no Grupo Controle e 82,3% no Grupo Intervenção (p=0,399). Para temas abordados após ação educativa, houve mais de 80% de acertos sobre alimentação, hidratação frequente, uso e função do imunossupressor. Entretanto, houve menos de 10% de acertos sobre higienização das mãos, contato com animais e aglomerações de pessoas. A utilização dos recursos áudio, visual e tátil do paciente ocasionou melhora na compreensão sobre cuidado com a pele (p=0,014).

**Conclusões:**

o nível de compreensão adquirido às recomendações para saúde foi apenas razoável.

## INTRODUÇÃO

A educação em saúde é considerada um dos caminhos que favorece a promoção de saúde por meio de intervenções educativas que auxiliam no desenvolvimento da responsabilidade individual e na prevenção de doenças. Tais ações são delineadas pelos profissionais da área da saúde de modo a possibilitar ao paciente não só o entendimento sobre o seu estado, mas também, a capacitação de tomar decisões relacionadas ao cuidado em saúde^(1)^.

O enfermeiro pode ser considerado catalisador de mudanças para promover a saúde, ao realizar intervenções educativas junto aos pacientes. Catalisar mudanças significa criar ambientes e configurações de intervenções educadoras que fortaleçam a participação e a apropriação dos indivíduos no protagonismo de seu próprio tratamento^(2)^. Na enfermagem, essas práticas direcionam a capacitação individual, visam facilitar e desenvolver habilidades pessoais que, por consequência, resultam no empoderamento do autocuidado, além de fortalecer o vínculo entre paciente e profissional^(2-3)^.

A intervenção educativa, individual ou grupal, fornecida de forma interativa, promove a aprendizagem e representa grande potencial para favorecer a adesão ao comportamento desejado no tratamento. Para isso, a estratégia educativa escolhida deve ser pautada no empoderamento da pessoa, a fim de torná-la responsável pela condução da situação de saúde, subsidiando adesão como foco para a prática do autocuidado^(3)^. A aprendizagem é definida como o processo que permite aos indivíduos modificar seu comportamento^(1)^.

O transplante de fígado é um procedimento cirúrgico complexo, que envolve cuidados permanentes devido ao risco de rejeição do enxerto. Para isso, é necessário aderir a mudanças no estilo de vida, como por exemplo, o rigor no uso medicamentoso e seu monitoramento nos hábitos higiênicos, na hidratação oral e nas condutas preventivas para infecção^(1)^.

Após o transplante de fígado, o indivíduo necessita de conhecimento para mudar alguns hábitos e melhorar sua qualidade de vida. Essa alteração comportamental inclui a adesão ao tratamento proposto depois do transplante, que envolve o novo regime medicamentoso, o comparecimento às consultas com a equipe de saúde, a realização de atividade física rotineira e atenção aos hábitos alimentares. É crucial que o paciente assuma a responsabilidade sobre o seu tratamento. O desconhecimento de condutas e de recomendações a serem praticadas exercem influência negativa no processo de adesão ao tratamento, tornando-se um risco para a evolução do funcionamento adequado do enxerto.

## OBJETIVOS

Avaliar o impacto da intervenção educativa na compreensão sobre as recomendações para saúde após transplante de fígado. A seguinte hipótese foi testada: o uso de intervenção educativa desenvolvida produz impacto positivo na compreensão sobre recomendações para saúde após o transplante de fígado.

## MÉTODOS

### Aspectos éticos

O presente estudo foi aprovado pelos Comitês de Ética em Pesquisa da Universidade Federal de Minas Gerais e da Santa Casa de Belo Horizonte, registrado no Registro Brasileiro de Ensaios Clínicos (ReBEC), sob o código RBR-454t52.

### Delineamento do estudo

Trata-se de um ensaio clínico, randomizado e prospectivo. Este estudo seguiu as orientações do *Consolidated Standards of Reporting Trials* (CONSORT)^(4)^ para apresentação de ensaios clínicos e foi registrado no Registro Brasileiro de Ensaios Clínicos (REBEC) sob o código de registro RBR-454t52.

### Período, local e população

O estudo foi desenvolvido no período de janeiro de 2016 a julho de 2019, em dois hospitais gerais e públicos de Belo Horizonte que possuem programa de transplante de fígado cadastrado no Sistema Nacional de Transplantes. Foram incluídos pacientes com idade igual ou superior a 18 anos, hospitalizados para realização do primeiro transplante hepático, com condições clínicas para receberem as recomendações para saúde destinadas ao autocuidado domiciliar após alta hospitalar.

### Critérios de seleção e formação da amostra

A randomização ocorreu no dia da realização do transplante por meio de sorteio. O recrutamento foi realizado pelo pesquisador após realização do transplante, um dia após a transferência do paciente da unidade de terapia intensiva para a unidade de internação. Na abordagem inicial, os pacientes que aceitaram participar da ação educativa assinaram o termo de consentimento informado e foram incluídos no estudo. O Consentimento Livre e Esclarecido foi obtido de todos os indivíduos envolvidos no estudo por meio escrito. O tamanho da amostra, determinado com uso do nível de significância de 5% e estimativa da proporção de 0,5, apontou a necessidade de no mínimo 31 pacientes/grupo. No total, 118 pacientes foram submetidos ao transplante de fígado nas instituições de saúde no período do estudo. Dos transplantados, 68 preencheram os critérios de inclusão e foram distribuídos para um dos dois braços do estudo: aqueles que receberam as recomendações para saúde acrescida de recomendações específicas sobre as drogas imunossupressoras, utilizando os recursos visual, auditivo e tátil do paciente na ação educativa foram alocados no grupo de intervenção (GI; n=34); e o grupo controle (GC; n=34) foi composto por aqueles que receberam as recomendações para saúde utilizando os recursos auditivo e visual do paciente na ação educativa convencional, consolidada na instituição. A Figura 1 mostra o diagrama do CONSORT, com o fluxo dos participantes durante as fases do estudo.

**Figura 1:** Fluxograma da formação da amostra do estudo

### Instrumentos utilizados

Na ação educativa convencional do GC, elaborou-se um material impresso, explicativo e validado nas instituições de saúde, de uso consolidado para alta hospitalar dos pacientes transplantados de fígado. Para o GI, utilizou-se o material produzido pela autora (um folder educativo, de encadernação espiral, com 15 figuras ilustrativas em formato JPEG impressos em folha A3), não revalidado por ser a reorganização das informações do impresso institucional acrescido de cores, figuras e detalhes atrativos.

### Intervenção

A ação educativa ocorreu no momento da alta hospitalar, momento em que cada um dos pacientes transplantados recebeu as mesmas recomendações para saúde específicas para o autocuidado. As recomendações para saúde foram fornecidas de formas distintas para o GI e GC. Elas englobaram alimentação, tomada dos medicamentos nos horários e doses corretas, não tomar a dose antes da realização de exames séricos laboratoriais para controle da dosagem de imunossupressores. Também foram recomendadas condutas a serem evitadas, tais como aglomeração de pessoas, contato com animais, contato com recém-nascidos que foram imunizados com vacina Sabin e com pessoas doentes. Além disso, uso de máscara nos primeiros 2 meses pós-transplante, prática de exercícios físicos leves tais como caminhadas, ingestão de água com frequência, aplicação de creme hidratante diariamente e comparecimento às consultas ambulatoriais nas datas estipuladas também foram enfatizados.

A ação educativa efetuou-se da seguinte maneira nos grupos: O GC recebeu as recomendações pelos enfermeiros integrantes da Equipe de Enfermagem para alta hospitalar, na unidade de internação das instituições de saúde do estudo. O material explicativo próprio das instituições de saúde foi impresso contendo uma página frente e verso. Na frente, havia instruções do mapa de medicamentos, ilustrado com os horários das tomadas e, no verso, recomendações de saúde por escrito, conforme apresentado na Figura 2.

**Figura 2:** Peça gráfica utilizada na ação educativa do Grupo Controle

A operacionalização da ação educativa do GC iniciou com a transcrição pelo enfermeiro dos medicamentos prescritos pela equipe médica, no mapa de medicamentos. Em seguida, o profissional dirigiu-se ao quarto do paciente e efetivou a ação educativa em dois momentos, conforme Figura 2. No primeiro, empregou-se o recurso auditivo do paciente com a realização da leitura dialogada, pelo enfermeiro, juntamente às recomendações escritas no verso do impresso. No segundo momento, mobilizou-se o recurso visual do paciente, com a entrega do impresso, com um mapa de medicamento transcrito. O enfermeiro realizou, junto ao paciente, a conferência dos medicamentos com as respectivas prescrições, identificando-os no mapa de medicamentos transcritos e mostrando as doses a serem administradas pelo paciente em domicílio. Já o GI recebeu as recomendações do enfermeiro-pesquisador, a partir do material utilizado, que incluía um folder impresso acrescido do mapa de medicamentos, bem como um material encadernado com figuras ilustrativas correspondentes a todas as recomendações de saúde além das específicas dos medicamentos imunossupressores, de acordo com a Figura 3.

**Figura 3:** Peça gráfica utilizada na ação educativa do Grupo Intervenção

A operacionalização da ação educativa do GI realizou-se em três momentos, utilizando-se os recursos auditivos, visuais e táteis dos pacientes, de acordo com a Figura 3. O primeiro e segundo momentos aconteceram de modo concomitante, empregou-se o recurso auditivo do paciente, com a leitura dialogada do folder educativo, e o recurso visual do paciente, pela apresentação das figuras ilustrativas encadernadas referentes às recomendações do folder. No terceiro momento, usou-se o recurso tátil do paciente, por meio da manipulação de medicamentos, a fim de reconhecê-los (isto é, observando formatos e texturas), seguido da confecção do mapa contido no folder. O paciente realizou, com auxílio do enfermeiro, a transcrição dos medicamentos prescritos pelo médico, bem como marcações dos horários e doses a serem administradas. O profissional também auxiliou o paciente na identificação dos medicamentos em mãos e sua localização no mapa transcrito por ele.

### Coleta de dados

A coleta de dados ocorreu 15 dias após alta hospitalar, no ambulatório em que o paciente foi entrevistado(a). Foram utilizados dois questionários elaborados pela autora, o primeiro com informações sobre as variáveis sociodemográficas e clínicas descritas na literatura como relevantes (idade, sexo, cidade de moradia, escolaridade, anos de estudo, indicação ao transplante, data de inscrição em lista de espera, data do transplante e data da alta hospitalar), preenchido pelo pesquisador por meio de entrevista e consulta ao prontuário. O segundo questionário visou estimar a compreensão das recomendações e foi composto por 13 questões de múltipla escolha, referentes às recomendações recebidas durante a alta hospitalar. O questionário foi entregue ao participante para preenchimento (autoadministrado) por um avaliador externo, que não possuía conhecimento sobre as ações educativas e objetivos do estudo. A cada afirmativa, o entrevistado assinala somente uma resposta como: totalmente verdadeira, parcialmente verdadeira, não sabe dizer, parcialmente falsa e totalmente falsa. O roteiro avaliou o nível de compreensão por parte do paciente sobre recomendações para saúde recebidas durante a alta hospitalar e vivenciadas por 15 dias em domicílio. A pontuação total indica o número de itens que foram respondidos corretamente (1 ponto para cada acerto), sendo possível obter de 0 a 13 pontos. Após somatório dos pontos obtidos, foi realizada análise de concordância entre a resposta do entrevistado e as orientações fornecidas durante a alta hospitalar. A seguir classificou-se o nível de compreensão da seguinte maneira: Excelente - para entrevistados que obtiveram de 10 a 13 pontos; razoável - para os que obtiveram de 6 a 9 pontos; ruim - para os que obtiveram de 3 a 5 pontos; e péssimo - para aqueles abaixo de 3 pontos. Além da classificação do nível de compreensão do entrevistado, também houve a investigação, inscrita ao final do questionário, acerca da opinião sobre o impacto das recomendações recebidas durante a alta hospitalar, em atendimento às necessidades na vivência domiciliar. Isso ocorreu por meio da resposta de uma questão de múltipla escolha de cunho perceptivo, a qual classificou recomendações recebidas como: insuficiente, adequada ou excelente. Cada entrevistado recebeu um código para garantia do seu anonimato no estudo.

### Tratamento e análise dos dados

Realizou-se a análise estatística pelo *Software Package for Social Science* (SPSS) versão 20.0 para Mac (SPSS Inc., Chicago, Illinois, EUA). Em um primeiro momento, procedeu-se à análise descritiva dos dados por meio de tabelas de frequência para variáveis nominais e relativos a variáveis quantitativas, sumarizadas na forma de média, mediana, desvio-padrão, interquartil e percentuais. Em seguida aplicou-se o procedimento Kolmorov-Smirnov para distribuição de variáveis quantitativas. No segundo momento, realizou-se teste Qui-quadrado para a comparação entre GC e GI para variáveis nominais. O teste Qui-quadrado comparou a compreensão e detectou diferenças significantes entre variáveis nominais. O teste T de *student* foi realizado para dados com distribuição normal e o teste Mann-Whitney para dados com distribuição não normal. Os resultados foram apresentados com média mais ou menos (±) desvio-padrão para variáveis numéricas com distribuição normal, mediana e intervalo interquartil para variáveis numéricas com distribuição não normal. Os dados foram distribuídos e relacionados em tabelas.

## RESULTADOS

Entre os 68 pacientes que participaram do estudo, 46 (67,6%) eram homens e 22 (32,4%) eram mulheres. A mediana de idade ao transplante foi de 56,5 anos (IIQ = 63,75). Trinta e sete pacientes (54,4%) eram procedentes da região metropolitana de Belo Horizonte e 31 (45,6%) vieram do interior de Minas Gerais. A média de anos de estudo era de 8,5 (±4,0), sendo que 34 (50,0%) possuíam a escolaridade até o ensino fundamental e 34 (50,0%) ensino médio ou mais. A média do tempo de hospitalização (a partir do dia do transplante até o dia de alta hospitalar) foi de 20 dias (±12,6), sendo a mediana de tempo de espera por um órgão de 63,0 dias (IIQ = 120,0). As indicações ao transplante foram agrupadas resultando em 32 (47,1%) por doença hepática parenquimatosa, 19 (27,9%) por neoplasia, 5 (7,4%) por doença de origem biliar, 3 (4,4%)por doença de origem autoimune, 1 (1,5%) por hepatite fulminante e 8 (11,8%) por outras doenças que foram reunidas por falta de similaridade com as doenças dos grupos previamente estabelecidos, sendo elas: 2 Doença de Caroli, 3 Síndrome Budd Chiari, 1 Amiloidose familiar, 1 Deficiência Alfa 1 Antitripsina e 1 Hemocromatose hereditária.

Com relação aos índices de acertos da amostra às recomendações fornecidas sobre: alimentação, uso correto do medicamento imunossupressor, funcionalidade dos imunossupressores e recomendação de hidratação oral frequente, os pacientes apresentaram mais de 80% de acertos. Higienização das mãos e condutas a serem evitadas - como aglomerações e contato com animais - expressaram menos de 10% de acertos (Tabela1).

Na análise comparativa das respostas corretas entre os grupos em estudo, participantes do GC e do GI obtiveram mais de 80% de acertos nas questões referentes à alimentação, uso correto do medicamento imunossupressor, função da medicação imunossupressora e hidratação oral. Os participantes do GI obtiveram mais acertos, entre 40% a 70%, nas recomendações sobre atividade física, não tomar medicação imunossupressora antes da coleta de sangue e condutas a serem evitadas como: contato com pessoas doentes e recém-nascidos vacinados com a Sabin, além dos cuidados com a pele. A recomendação de cuidados com a pele foi respondida erroneamente por 40 (58,8%) dos 68 participantes do estudo, na comparação entre os grupos somente esta recomendação apresentou diferença estatística (p = 0,014), sendo os acertos mais frequentes no GI (Tabela 1).

**Tabela 1:** Avaliação do questionário de compreensão das orientações de saúde fornecidas no momento da alta hospitalar de janeiro de 2016 a julho de 2019, Belo Horizonte, Minas Gerais, Brasil

Questões sobre as orientações fornecidas	Am† (n=68)		||GC (n=34)		¶GI (n=34)		Valor de p^*^
	A‡ n, %	E§ n, %	A‡ n, %	E§ n, %	A‡ n, %	E§ n, %	
1) O Sr. (a) deverá seguir as orientações da nutricionista, lembrando que não pode ingerir frutas com casca e nem ingerir legumes crus.	63 92,6	5 7,4	33 97,0	1 3,0	30 88,3	4 11,7	0,163
2) O Sr. (a) deve sempre lavar as mãos e friccionar álcool.	4 5,9	64 94,1	1 3,0	33 97,0	3 8,9	31 91,1	0,303
3) O Sr. (a) deve fazer musculação 02 vezes na semana nos primeiros três meses após o transplante.	38 55,9	30 44,1	17 50,0	17 50,0	21 61,7	13 38,3	0,329
4) O Sr. (a) deve tomar todos os medicamentos certos com a dose correta e o horário correto conforme a prescrição.	67 98,5	1 1,5	33 97,0	1 3,0	34 100	0 -	0,314
5) Antes de colher o sangue para exame o Sr. (a) pode tomar todos os medicamentos.	37 54,4	31 45,6	17 50,0	17 50,0	20 58,8	14 41,2	0,465
6) O Sr. (a) sabe que os medicamentos da ISS^**^ servem para evitar a destruição do órgão transplantado pela defesa do organismo.	63 92,6	5 7,4	31 91,1	3 8,9	32 94,1	2 5,9	0,642
7) Os medicamentos ISS^**^ podem causar aumento da glicose, dor de cabeça, tremores, insônia e diarreia, caso isso acontecer, o Sr. (a) deve informar nas consultas.	53 77,9	15 22,1	27 79,4	7 20,6	26 76,4	8 23,6	0,770
8) O Sr. (a) deve evitar lugares fechados e com muitas pessoas.	4 5,9	64 94,1	1 3,0	33 97	3 8,9	31 91,1	0,303
9) É permitido o contato com as crianças recém-nascidas após a vacina da Sabin e pessoas doentes.	47 69,1	21 30,9	21 61,7	13 38,3	26 76,4	8 23,6	0,189
10) É aconselhável o Sr. (a) ajudar a cuidar de animais, principalmente gatos, cães e aves, desde que o Sr. (a) não toque neles.	6 8,8	62 91,2	4 11,7	30 88,3	2 5,9	32 94,2	0,393
11) É importante que o Sr. (a) use a máscara o tempo todo.	27 39,7	41 60,3	13 38,2	21 61,8	14 41,2	20 58,8	0,804
12) É recomendado ao Sr. (a) beber muito líquido, aproximadamente 03 litros por dia.	59 86,8	9 13,2	28 82,4	6 17,6	31 91,1	3 8,9	0,283
13) Para manter a pele hidratada o Sr. (a) pode usar diariamente creme.	28 41,2	40 58,8	9 26,4	25 73,5	19 55,8	15 44,2	0,014

* Teste Qui-quadrado; †Am - Amostra; ‡ A - Acerto; § E - Erro; ||GC - Grupo Controle; GI - Grupo Intervenção;

** ISS - Imunossupressores.

Em relação à análise comparativa das respostas incorretas entre os grupos em estudo, embora o GC tenha apresentado mais erros no questionamento sobre recomendações de higienização das mãos e sobre a conduta de evitar aglomerações de pessoas e o GI tenha errado mais frequentemente sobre a conduta de evitar contato com animais, não houve diferença significativa nas respostas entre os grupos estudados (Tabela 1).

Em relação à análise de concordância entre a resposta do entrevistado às recomendações fornecidas durante alta hospitalar, dos 68 pacientes, 53 (77,9%) apresentaram razoável nível de compreensão. Sendo que 66 deles (97,0%) opinaram como excelentes e adequadas as informações recebidas. (Tabela 2).

**Tabela 2:** Análise comparativa entre Grupo Controle e Grupo Intervenção do nível de compreensão e opinião dos participantes no período de janeiro de 2016 a julho de 2019, Belo Horizonte, Minas Gerais, Brasil

Variáveis	Categorias	Amostra n = 68 (n, %)	Grupo Controle n=34 (n, %)	Grupo Intervenção n=34 (n, %)	Valor de p^*^
Nível de compreensão dos participantes às orientações recebidas	Excelente Razoável Ruim	03 (4,5) 53 (77,9) 12 (17,6)	01 (3,0) 25 (73,5) 08 (23,5)	02 (5,9) 28 (82,3) 04 (11,8)	0,399
Opinião do paciente sobre a orientação recebida	Adequado Excelente Insuficiente	19 (27,9) 47 (69,1) 02 (3,0)	07 (20,6) 26 (76,4) 01 (3,0)	12 (35,3) 21 (61,7) 01 (3,0)	0,397

* *Teste Qui-quadrado.*

Não houve diferença significativa no nível de compreensão ao comparar os grupos do estudo (p=0,399) (Tabela 2).

## DISCUSSÃO

A ação educativa ocorreu com recursos didáticos e apresentação do material de formas distintas. Cada intervenção ocorreu durante a alta hospitalar de pacientes que se submeteram ao transplante de fígado, possibilitando um momento esclarecedor para o paciente e seus familiares. Os achados deste estudo chamam a atenção para o impacto variado em relação à compreensão dos participantes, evidenciado por respostas com índices extremos de erros e acertos.

Percebe-se o impacto positivo das recomendações de alimentação, hidratação oral frequente, funcionalidade e uso de imunossupressor, que se destacaram pelos elevados índices de respostas corretas obtidas. Importante notar que as recomendações foram vivenciadas pelo paciente ainda no período de permanência hospitalar (que durou em média 20 dias) e, consequentemente, foram temas abordados diariamente pela equipe de saúde. Portanto, o contato prévio pode ter facilitado a compreensão e a manutenção de condutas em domicílio. Entretanto, a recomendação sobre higienização das mãos não apresentou impacto relevante pelas respostas obtidas. Possivelmente, o uso frequente de álcool no âmbito hospitalar pode ter provocado certa confusão no paciente sobre a maneira correta de higienizar as mãos em domicílio.

Na ação educativa sobre essa recomendação, alterações podem ser necessárias durante o ensino em saúde para reforçar o hábito de lavagem das mãos com água e sabão, tanto para o paciente como também para família e visitantes. No período de hospitalização, pode surgir o desejo de melhora da saúde, que faz com que o paciente crie expectativas que influenciam diretamente na sua recuperação^(5)^. As pessoas são capazes de armazenar muito mais informações quando as veem e ouvem. A repetição de informações é citada como importante forma de memorização, o que nos leva a repensar as estratégias de ensino-aprendizagem atualmente empregadas^(6)^.

Outras recomendações não apresentaram impacto, a exemplo da recomendação de evitar aglomerações de pessoas e contato com animais (principalmente gatos, cães e aves). Elas foram evidenciadas por menores índices de acerto por parte dos participantes. Observou-se, que nesses dois temas, também será necessário modificar a forma de ensino, reforçando exemplos de situações cotidianas que devem ser evitadas, como a visita a templos religiosos, estádios, supermercados, shoppings, agências bancárias ou compartilhamento de cama ou sofá com animal doméstico. É preciso também enfatizar aspectos negativos da proximidade com animais, como possibilidade de transmissão de zoonoses por meio do contato com fezes e saliva do animal^(7)^.

No presente estudo, um ponto relevante a ser destacado foi o impacto positivo no grupo que recebeu recomendações utilizando a associação dos recursos auditivos, visuais e táteis dos pacientes, que alcançaram o nível de compreensão de 82,3% dos participantes. Destaca-se, nesse grupo, a melhor compreensão sobre a recomendação do cuidado cutâneo diário com a aplicação de creme hidratante (p=0,014).

Na Espanha, um estudo evidenciou como benéficas as medidas educacionais realizadas após o transplante de fígado, por meio da associação de recursos verbais, escritos e audiovisuais como estratégias de ensino-aprendizagem sobre hábitos de vida saudáveis, o que se corrobora com o presente estudo. Segundo os autores, a associação desses recursos é mais eficaz do que apenas utilizar o recurso verbal^(8)^.

No entanto, o presente estudo detectou deficiência na compreensão sobre a rotina de cuidados com a pele, uma vez que 58,8% dos pacientes responderam erroneamente sobre o uso diário de creme hidratante na pele. Os cuidados dermatológicos são recomendados porque os receptores de transplante de órgão apresentam maior risco para infecções e câncer de pele. O regime imunossupressor facilita cerca de 70% das infecções cutâneas graves que aparecem durante os primeiros 3 meses após o transplante. É recomendado que receptores de transplante de órgãos sólidos desempenhem papel ativo na prevenção de complicações cutâneas. As intervenções educacionais podem resultar em melhor informação para essa população específica e favorecer o tratamento imediato, com melhores resultados clínicos^(9)^.

Quanto à prevenção do câncer de pele, um estudo mostrou que existe a tendência de o gênero masculino não aderir à aplicação de protetor solar na pele de forma rotineira e regular como autocuidado. Já as mulheres usam protetor solar no dia a dia com mais frequência do que homens^(10)^. Tal comportamento revela um problema a ser combatido por meio de elaboradas campanhas para fotoproteção, com a finalidade de atingir o público masculino de forma mais eficiente e de estimular o hábito de aplicação de protetor solar^(11)^. Homens parecem menos preocupados com os efeitos nocivos da radiação ultravioleta do que as mulheres, que usam filtro solar com mais frequência, provavelmente devido ao maior senso de cuidado com sua saúde e estética^(12)^.

Desse modo, se por um lado os resultados apontaram para melhor compreensão do autocuidado dermatológico dos participantes do GI, por outro, chama a atenção o hábito de cuidado com a pele, que difere nos gêneros em nossa sociedade. O conhecimento de tal comportamento é de significativa importância para formulação de intervenções concisas, robustas e direcionadas a essa demanda identificada.

A avaliação da ação educativa identificou nível de compreensão razoável dos participantes no presente estudo ao utilizar material didático com folder explicativo e ilustrado, impresso explicativo, mapa de medicação, além da presença do profissional educador (enfermeiro), que usou de recursos auditivos, visuais e táteis dos pacientes para executar a ação. O enfermeiro tem papel fundamental na implementação de ações educativas, que constituem medidas relativamente simples e de baixo custo, porém eficazes^(13)^. Nos temas que não alcançaram níveis adequados de compreensão o estudo, foi útil a identificação da necessidade de abordagens diferenciadas. A literatura sugere a incorporação do emprego de tecnologias para intervenção educacional, que pode ser uma opção utilizada pelos centros transplantadores de maneira útil e eficaz^(14-15)^. O uso de aplicativos para educação de pacientes é recente, no entanto, o emprego de mensagens, vídeos, imagens e animações são recursos que apresentam resultados efetivos de intervenção quanto ao conhecimento e cuidados^(16)^.

Por se tratar de intervenção educacional, era esperado que o nível escolar pudesse comprometer a compreensão do paciente às recomendações fornecidas. O estudo mostrou que 77,9% dos participantes apresentaram razoável compreensão, independentemente do grau de escolaridade. Outros estudos corroboram com este achado, apontando, ainda, que a percepção e a satisfação individual são mais influenciadoras na adesão ao tratamento do que o nível de instrução^(17-18)^.

Este estudo evidenciou que 69,1% dos participantes avaliaram como excelentes as recomendações ministradas, no atendimento de suas necessidades. Mesmo com o nível de compreensão razoável dos participantes, este achado traduz o estado de satisfação dos entrevistados perante as informações que subsidiaram seu autocuidado domiciliar.

### Limitações do estudo

É importante ressaltar que o presente estudo apresenta como limitação a ausência de resultados de médio e longo prazo. Entretanto, essa limitação não comprometeu a qualidade da avaliação e os resultados obtidos, os quais pretendiam verificar o impacto precoce no cumprimento das recomendações.

### Contribuições para a Área

Nossa contribuição mais relevante foi demonstrar que a preparação para o autocuidado do paciente pode ser realizada durante o período de hospitalização, com a participação da equipe de saúde assistente, oportunizando a vivência experimentada pelo paciente e familiares.

Acreditamos que quanto maior o número de abordagens o enfermeiro implementar no processo de educação do paciente e familiares, em suas diferentes formas, melhor será a continuidade do seu autocuidado. Entretanto, para mensurar mais adequadamente esse efeito, outros estudos prospectivos específicos devem ser realizados.

## CONCLUSÕES

A ação educativa de ensino para saúde, realizada no momento da alta hospitalar e com foco na prática do autocuidado, alcançou nível razoável de compreensão dos pacientes transplantados. A intervenção educativa desenvolvida produziu impacto positivo na compreensão das recomendações para saúde após transplante de fígado.
